# Symposia on Plant (Protein) Phosphorylation

**DOI:** 10.3389/fpls.2012.00201

**Published:** 2012-08-31

**Authors:** Sacco C. de Vries

**Affiliations:** ^1^Laboratory of Biochemistry, Wageningen UniversityWageningen, Netherlands

From September 14 to 16, 2011 the 12th symposium on Plant Protein Phosphorylation was held in Tübingen, Germany. The topic is as broad as the name suggests and covers all aspects of this important means of protein modification in plants. I have had the pleasure of attending the 2007 and the 2011 symposia. The interesting concept behind these meetings is to hear about the same biochemical mechanism operative in a multitude of experimental systems. The meetings are quite informal and present an excellent mix ranging from technology to biochemical experience and novel findings and tools.

The two-and-a-half-day program was divided into five double sessions: biotic interactions, hormone signaling, abiotic interactions, Mitogen Activated Protein Kinase (MAPK), and Ca^++^ pathways and phosphoproteomics. It was hosted by the Zentrum für Molekularbiologie der Pflanzen (ZMBP) and the organizing committee chaired by Klaus Harter.

In the biotic interactions session the role of phosphorylation in Pathogen-Triggered Immunity (PTI) was the central topic. Several speakers discussed differential phosphorylation events after turning on the PTI pathways. The first presentation was by Yuelin Zhang (Beijing) who discussed recent work on identifying targets of the MAPK pathway using a suppressor screen. To what extent cross talk between growth and PTI pathways exists at the level of the receptors was discussed by Cyril Zipfel (Norwich). Important points raised were whether the dynamics of receptor activation as observed in brassinosteroid receptors are directly applicable to the non-Arginine Aspartic Acid (RD) type of kinases that seem to feature in most PTI pathways. A very intriguing report was made by Iris Jarsch (Munich), suggesting a role for remorins to sequester receptors in small membrane micro domains. Looking in the very first events of membrane receptor activation, it appears that changes in heteromeric state of Flagellin Sensing 2 (FLS2) and BRI1 Associated Kinase (BAK1) can be seen a mere few seconds after ligand application (Birgit Schulze, Basel). Speed was also the topic of a presentation from the host (Klaus Harter, Tübingen), showing evidence for direct activation of plasma membrane adenosine triphosphatases (PM ATPases) by the BRI1 receptor. Down-regulation by endocytosis of the FLS2 receptor, negative control elements such as phosphatases appear to play more and more important roles in controlling receptor activity and sensitivity (Antje Heese, Columbia; Roda Niebergall, Norwich) while genes have been identified that modulate the calcium signature of the PTI response (Justin Lee, Halle).

A much-explored theme in the signaling session was to isolate receptor-interacting proteins by direct pull downs or yeast interaction screens. This was used in approaches by Sorina Popescu (Ithaca) investigating reticulon-like proteins that regulate trafficking and activity of FLS2 and Sacco de Vries (Wageningen) looking at the interaction properties of members of the Somatic Embryogenesis Receptor-like Kinase (SERK) family of non-ligand binding coreceptors that includes BAK1(SERK3). Other work dealt with control of cellulose synthase by phosphorylation (Martine Gonneau, Versailles), subcellular relocalization of Pin-formed (PIN) proteins through phosphorylation by PINOID kinases (Eike Rademacher, Leiden) and several talks on the direct control of abscisic acid (ABA) signaling by Protein Phosphatase 2C (PP2C) enzymes (Erwin Grill, München; Rainer Hedrich, Würzburg) including interesting kinases that lack the conserved lysine in the catalytic domain called With No K (WNK)s; Esther Görlich, Heidelberg). Using an alternative approach by comparing phosphorylation patterns in the presence or absence of a specific inhibitor of the salicylic acid (SA) response-mediating Casein Kinase 2 (CK2), an SA-induced phosphorylated p23 protein was identified that may act in a Heat Shock Protein (HSP) 90 complex (Stefano d’Allessandro, Padua). Clearly, molecular approaches using phospho-site mutants are generally applied to unravel functional relevance of the proteins under investigation.

The next session was devoted to abiotic signaling and started with Jay Thelen (Columbia) introducing a number of nice generally applicable tools to discover phosphorylation sites (MUSite^1^) and Kinase Clients including the novel peptide/mass spectrometry-based Kinase Client assay (KiC assay). He also provided many examples of the genome-wide data sets available in the Plant Protein Phosphorylation Database at Columbia^2^. Response to Reactive Oxygen Species involves so-called Cysteine-rich Receptor-like Kinases (RLKs) that may be the direct sensors of ozone. Intriguingly, a small peptide derived from a protein called Grim Reaper (GRI) induces cell death and may function in the ROS pathway (Jaakko Kangasjärvi, Helsinki). Claudia Jonak (Vienna) discussed the role of the *Arabidopsis* S-phase Kinase Associated Protein 1 (SKP1)-like 5 (ASK5) kinase in linking adaptive regulation of the redox balance via control of the phosphorylation status of the pentose phosphate entry enzyme glucose 6-phosphate dehydrogenase (G6PD). To enlarge the scope of the downstream changes in phosphorylation patterns upon stress induction several talks (Maik Böhmer, La Jolla; Kazuo Shinozaki, Tsukuba; Michael Sussman, Madison) used ABA signaling as a starting point to describe genome-wide changes in the phosphoproteome. The challenges in these large-scale projects are obviously the reliability of the predicting software, the coverage obtained, and the required biological verification of the changes encountered. The last two talks in this session were given by Markus Teige (Vienna) and Thomas Guérinier (Orsay). Teige described an elegant approach to purify kinases that give specific changes to target transcription factors, in this case a member of the basic region Leucine Zipper (bZIP) family involved in metabolic reprogramming. This approach also highlighted the temporal and site specific dynamic changes in the phosphorylation pattern. A different set-up was used by Guérinier to identify peptide-based kinase assays to identify novel modulating kinase effectors such as nucleotides using Sucrose Non-fermenting 1 Related Protein Kinase 1 (SnRK1) as a model.

The following session dealt with a series of experimental systems used to gain more insight into the subcellular specificity in signaling systems. The calcium-dependent protein kinases (CDPKs) were presented by Tina Romeis (Berlin) as mediators and perhaps integrators of ABA and Pathogen Associated Molecular Pattern (PAMP) responses. Here *in vivo* phosphoproteomics was used to identify direct targets of the CDPKs. How calcium is sensed intracellularly by Calcineurin B-like Proteins (CBLs) and associated CBL-Interacting Protein Kinases (CIPKs) was discussed by Jörg Kudla (Münster) and showed a highly complex signaling network present at the PM and capable of decoding local changes in calcium and effecting directly sodium and potassium channels. From the same lab and in association with the group of Harter (Tübingen), Kenji Hashimoto presented results showing that the CBL1/CIPK1 pair acts as an important switch in translating light signaling into transcriptional changes. Then the MAPKs took center stage in talks by Souha Berriri (Evry) who used a gain-of-function screen for MAPKs first in yeast, as a tool to identify targets of the MAPK pathway. MAPK4 GOF mutants were then used to help delineate PTI and Effector-triggered Immunity (ETI) targets that run through the same MAPK cascade. The differences and similarities between human and plant innate immunity systems was discussed by Heribert Hirt (Evry) showing that *Arabidopsis* can be infected by *Salmonella typhimurium*, the major cause of food poisoning. Two kinases were identified that appear to act in a PTI pathway different from the MAPKs directly under control of PTI-related kinases. Genetic approaches were also employed by Roman Ulm (Geneva) having identified a phosphatase that mediates the UV-B stress response and by Marie Boudsocq (Harvard). In that work, CDPKs were identified that could mimick 22 amino acid flagellin fragment (FLG22) signaling and suggesting that alternative routes exist besides the classical MAPK pathway in PTI signaling. A constitutive CDPK under control of an inducible promoter was used to identify changes in the phosphoproteome (Guido Durian, Berlin).

The last sessions were devoted to technological advances in phosphoproteomics as being applied to a number of different systems. Themes that emerged were the necessity to use well-defined induction systems, the use of biological and technical replicates and the transient qualitative changes that could be captured.

Naoyuki Sugiyama (Tsuruoka) introduced Hydroxy Acid Modified Metal Oxide chromatography (HAMMOC) reported to increase the yield and specificity of phosphopeptides from complex digests. A number of examples were shown in both rice and *Arabidopsis* that clearly showed the improvement of these methods coupled to improved search algorithms. In particular tyrosine phosphorylation appears to be as widespread in *Arabidopsis* as in our own cells! The effects on the phosphoproteome that can be distinguished after nutrient uptake can be separated into early and late events, involving both different proteins and different phospho-sites. The tools are available through improved web-based applications (PhosPhAt^3^, Waltraud Schulze, Golm). The problems associated with quantitative phosphoproteomics were addressed by Frank Menke (Utrecht) with MAPK cascades as an example and using differential N14/N15 labeling in cell cultures. Borjana Arsova (Golm) combined a nitrogen starvation/re-supply experiment with membrane protein isolation and phoshoproteomics and identified a membrane kinase that responds to ammonium but not to nitrate. Differential labeling is mostly confined to cell cultures meaning that label-free quantification procedures are required when dealing with plants or plant organelles. Such methods were employed by Sacha Baginsky (Halle) to identify differentially phosphorylated proteins in *Arabidopsis* chloroplasts. Biological replicates and the use of mutants in crucial kinases such as Protein State Transition 8 (STN8) were employed to provide reliable data. Further improvements in these procedures were presented by Wolfram Weckwerth (Vienna) using better software prediction coupled to metal oxide purifications and by Pururawa Mayank (Zürich) using similar approaches but now to describe the pollen phosphoproteome. The last talk was given by Thomas Stratil (Münich) returning to the exciting remorin proteins (see Jarsch, also of the Thomas Ott Lab) of which one was also found to be important for *Medicago* root nodule development. It was directly associated with one of the symbiotic RLKs.

Fifty-six posters of excellent quality were on display in front of the auditorium during the entire meeting and formed the central point of discussion during the coffee and tea breaks. In the short space allotted to this report it is unfortunately not possible to describe them all (for abstracts see: Plant Protein Phosphorylation Symposium 2011. September 14 to 16, 2011. Program and Abstracts. University of Tübingen, Auf der Morgenstelle 3, Tübingen, Germany).

## Trends and Perspectives

The improvements of the metal oxide-based phosphopeptide purification procedures, improved sensitivity of the Mass Spectrometer (MS) and better software have all resulted in a situation that *in vivo* determination of particular phospho-sites is feasible. An example of that can be found in a recent publication of the Jenny Russinova Lab (Ghent; Gudesblat et al., 2012) where the change in phosphorylation status of a single biologically important residue of the transcriptional regulator SPEECHLESS was recorded. Dephosphorylation of Ser65 of this protein occurred in response to activation of the brassinosteroid signaling pathway through its main receptor BRI1 and was performed using intact *Arabidopsis* seedlings as starting material. Clearly, label-free quantification approaches have reached maturity.

Realization that most phosphorylation changes upon or during activation of a pathway are transient and also multiple will require time-lapse recording experiments at the second and minute time scale. So far this is only possible in single or few protein analyses; for an example of such an analysis using the Epidermal Growth Factor Receptor (EGFR; see Dengjel et al., 2007). It is not yet easy to see how such dynamic pictures can be applied to genome-wide analyses in the absence of labeling.

Efforts to visualize first general and next specific phosphorylation events as is now relatively common in mammalian signaling systems (EGFR; Verveer et al., 2000) will no doubt further increase our insights into the fascinating field of Plant Protein Phosphorylation.

## Footnotes

^1^http://musite.sourceforge.net

^2^http://www.p3db.org

^3^http://phosphat.mpimp-golm.mpg.de
